# Morphologies, dimensions and targets of gastric nitric oxide synthase neurons

**DOI:** 10.1007/s00441-022-03594-0

**Published:** 2022-02-11

**Authors:** Madeleine R. Di Natale, Billie Hunne, Jamie J. M. Liew, Linda J. Fothergill, Martin J. Stebbing, John B. Furness

**Affiliations:** 1 Department of Anatomy & Physiology, University of Melbourne, Parkville, VIC 3010 Australia; 2 Florey Institute of Neuroscience and Mental Health, Parkville, VIC 3010 Australia

**Keywords:** Stomach, Nitric oxide, Nitrergic neurons, Inhibitory neurons, Gastric motor neurons, Enteric nervous system

## Abstract

We investigated the distributions and targets of nitrergic neurons in the rat stomach, using neuronal nitric oxide synthase (NOS) immunohistochemistry and nicotinamide adenine dinucleotide phosphate (NADPH) diaphorase histochemistry. Nitrergic neurons comprised similar proportions of myenteric neurons, about 30%, in all gastric regions. Small numbers of nitrergic neurons occurred in submucosal ganglia. In total, there were ~ 125,000 neuronal nitric oxide synthase (nNOS) neurons in the stomach. The myenteric cell bodies had single axons, type I morphology and a wide range of sizes. Five targets were identified, the longitudinal, circular and oblique layers of the external muscle, the muscularis mucosae and arteries within the gastric wall. The circular and oblique muscle layers had nitrergic fibres throughout their thickness, while the longitudinal muscle was innervated at its inner surface by fibres of the tertiary plexus, a component of the myenteric plexus. There was a very dense innervation of the pyloric sphincter, adjacent to the duodenum. The muscle strands that run between mucosal glands rarely had closely associated nNOS nerve fibres. Both nNOS immunohistochemistry and NADPH histochemistry showed that nitrergic terminals did not provide baskets of terminals around myenteric neurons. Thus, the nitrergic neuron populations in the stomach supply the muscle layers and intramural arteries, but, unlike in the intestine, gastric interneurons do not express nNOS. The large numbers of nNOS neurons and the density of innervation of the circular muscle and pyloric sphincter suggest that there is a finely graded control of motor function in the stomach by the recruitment of different numbers of inhibitory motor neurons.

**Supplementary information:**

The online version contains supplementary material available at 10.1007/s00441-022-03594-0.

## Introduction

The movements of the stomach are controlled by enteric excitatory and inhibitory motor neurons, whose activities are co-ordinated through the vagus nerve. Studies of the pharmacology of inhibitory transmission and of the release of inhibitory neurotransmitters indicate that the inhibition is mediated by nitric oxide (NO), vasoactive intestinal peptide (VIP) and a transmitter acting through purine receptors (Fahrenkrug et al. 1978; Li and Rand 1990; Boeckxstaens et al. 1991, 1992; Gil et al. 2013), with NO appearing to play the major role (Desai et al. 1994; Idrizaj et al. 2021). Knock out of the synthesizing enzyme, neuronal nitric oxide synthase (nNOS), results in defective gastric emptying, primarily due to the loss of inhibitory control of the pyloric sphincter (Huang et al. 1993). The inhibitory neurons are responsible for vagally mediated relaxation of the stomach, which is inhibited both by nicotinic receptor blockers and by a block of nitrergic transmission (Martinson 1965; Desai et al. 1991; Idrizaj et al. 2021). Nitric oxide synthase (NOS) is a nicotinamide adenine dinucleotide phosphate (NADPH) oxidase, requiring NADPH as a proton donor for the conversion of arginine to citrulline and the production of NO (Knowles and Moncada 1994). Thus, the neurons can be localised by nNOS immunoreactivity or histochemically, using the NADPH diaphorase reaction (Young et al. 1992; Schemann et al. 1995; Brookes et al. 1998).

In the small intestine, nNOS is in both inhibitory muscle motor neurons and interneurons, with the interneurons, but not the motor neurons, being cholinergic (Costa et al. 1996; Li and Furness 2000). In the stomach, populations of myenteric neurons containing nNOS and those with cholinergic markers, notably choline acetyltransferase (ChAT), do not overlap (Schemann et al. 1995; Brookes et al. 1998), suggesting that ChAT/nNOS interneurons are not present, despite there being evidence for the presence of nitrergic interneurons (Schemann and Schaaf 1995).

Retrograde labelling of myenteric neurons using DiI crystals placed on the circular or longitudinal muscle indicates that the motor innervation to these layers in the fundus and corpus comes from cell bodies in the myenteric ganglia in guinea-pigs (Brookes et al. 1998; Pfannkuche et al. 1998; Michel et al. 2000; Schemann et al. 2001). The myenteric origin of the circular muscle innervation has been confirmed by denervation surgery in dogs (Furness et al. 1991). There is a polarity in the motor neuron supply, the inhibitory neurons projecting anally before supplying circumferentially-oriented branches to the muscle, and the excitatory neurons projecting orally before branching in the gastric corpus (Brookes et al. 1998).

In summary, both structural and functional data indicate that gastric nNOS neurons supply the external muscle, but whether there are also nNOS interneurons, or nNOS neurons that supply other targets, is not resolved. In the present paper, we have examined the distributions of nNOS neurons and their terminals throughout the rat stomach in order to answer these questions.

## Methods

### Tissue sources

All the procedures were approved by the Florey Institute of Neuroscience and Mental Health Animal Ethics Committee (approval 21–010) or by the University of Melbourne Animal Ethics Committee (approval 10272). Rats were supplied with food and water ad libitum prior to any experiments. Stomach samples were collected from female and male Sprague Dawley (SD) rats, 6–10 weeks old, 161–240 g for females and 221–382 g for males.

### NADPH diaphorase histochemistry

NADPH diaphorase (NADPHd) histochemistry, based on a previously published method (Young et al. 1992), was investigated in whole mounts from 4 rats. All the procedures were at room temperature (RT) unless otherwise specified. Rats were deeply anaesthetised by isoflurane inhalation followed by decapitation, and then stomachs were removed and placed into PBS (phosphate-buffered saline: 0.15-M NaCl in 0.01 M sodium phosphate buffer, pH 7.2) containing nicardipine (1 µM; Sigma-Aldrich, Sydney, Australia) before being opened along the greater curvature and stretched and pinned to a balsa board, mucosal side down, for fixation in 4% formaldehyde, in 0.1-M phosphate buffer, pH 7.0, for 1 h at 4 °C. Fixative was removed by 3–6 − 10-min washes in PBS. Tissue samples were taken from the ventral fundus, corpus and antrum. Wholemounts of the longitudinal muscle and myenteric plexus were created by dissecting away the mucosa, submucosa and most of the circular muscle. Wholemounts of the submucosa were also prepared. Wholemounts were incubated with 0.3% Triton X-100 in PBS for 10 min followed by a 1 × 10 min-PBS wash, before being incubated in the NADPHd solution (0.1-M Tris buffer, pH 7.6, 0.5% Triton X-100, 0.25 mg/mL nitroblue tetrazolium, 1 mg/mL β-NADPH) (Sigma-Aldrich) for 10–25 min at 37 °C, while the colour reaction was monitored. Wholemounts were washed again with 3 × 10 min changes of PBS and mounted with Dako fluorescence mounting medium. Staining was assessed, and individual cell drawings were completed, using an Olympus camera lucida system, and samples were also imaged using a Mirax digital slide scanner (Zeiss, Sydney, Australia).

### Combined NADPH diaphorase and neuronal NOS staining

Wholemount preparations, that were fixed and dissected as above, were immunohistochemically stained with nNOS prior to NADPHd staining (Young et al. 1992). Wholemounts were incubated with a mixture of sheep anti-nNOS and human anti-Hu (see Supplementary methods for antibody details) in PBS with 0.25% Triton X-100, overnight at RT. The preparations were then washed 3 × 10 min in PBS before a 2-h incubation with Alexa Fluor^™^ secondary antibodies in PBS containing 0.25% Triton (see Supplementary methods). Samples received a further 3 × 10-min washes in PBS, before being mounted with buffered glycerol (90% glycerol in PBS). Selected ganglia were imaged using an AxioImager microscope or an LMS800 confocal microscope (Zeiss). The coverslip was then removed and the samples histologically stained for NADPHd, as described above. The samples were mounted with Dako fluorescence mounting media on slides, and the cells that were identified initially were re-imaged for NADPHd staining.

### Immunohistochemistry for neuronal NOS in wholemounts and cryostat sections

Fresh stomachs were collected from 8 rats that were deeply anaesthetised with an intraperitoneal injection of pentobarbital sodium (100 mg/kg), placed into PBS containing nicardipine (1 µM), and opened and pinned to a balsa board before being immersed in fixative (2% paraformaldehyde and 0.2% picric acid in 0.1-M sodium phosphate buffer, pH 7.0) overnight at 4 °C. Fixative was washed out with dimethyl sulfoxide (DMSO), 3 × 10 min, followed by PBS, 3 × 10 min, and tissue was stored in PBS-azide (0.1% sodium azide in PBS) at 4 °C until being prepared for wholemounts or sections.

Wholemounts were dissected to reveal the myenteric plexus, as described above. These preparations were blocked with normal horse serum (NHS) and Ttriton (10% NHS plus 0.1% Triton X-100 in PBS) at 37 °C for 2 h. Samples were then incubated with a mixture of primary antibodies, sheep anti-nNOS and human anti-Hu (see Supplementary methods), diluted in PBS-azide with 0.3% Triton X-100, 1% bovine serum albumen (BSA), and 1% NHS, overnight at 37 °C followed by 2 nights at RT. The preparations were then washed with PBS, 3 × 10 min before a 5-h incubation with mixtures of Alexa Fluro^™^ secondary antibodies diluted in PBS-azide with 0.3% Triton X-100 at 37 °C (Supplementary methods). Samples were washed in PBS, 3 × 10 min before being mounted with a Dako fluorescence mounting medium. Wholemounts were imaged using an AxioImager or an LMS800 confocal microscope.

These samples were used to determine the ratio of nNOS to Hu neurons in wholemount preparations from the fundus, corpus and antrum (8 preparations in total, from 2 female and 2 male rats). The number of nNOS immunoreactive (IR) nerve cells was counted in cohorts of 200 Hu positive neurons within each of these regions. The profile areas (μm^2^) of nNOS immunoreactive neurons were measured by manually drawing a line around the border of the cell body and dendrites using the graphics menu and measure functions in ZEN blue software (Zeiss) (see Supplementary methods).

For tissue that was to be prepared for cryostat sections and, after fixative was washed out, the regions of stomach to be sectioned were placed in 30% sucrose in PBS-sucrose-azide overnight at 4 °C followed by an overnight incubation in a mixture of OCT (optimal cutting temperature compound; Trajan Scientific and Medical, Ringwood, Australia) and PBS-sucrose-azide in a 1:1 ratio. Tissue blocks were embedded in 100% OCT medium and frozen in isopentane cooled by liquid nitrogen. Cryostat Sects. (12 μM) were cut and mounted onto SuperFrostPlus^®^ microscope slides (Menzel-Glaser; Thermo Fisher, Scoresby, Australia). Sections were air-dried for 1 h, blocked with NHS for 30 min at RT and then incubated in a mixture of primary antibodies: sheep anti-nNOS and rabbit anti-α smooth muscle actin (αSMA), diluted in PBS-azide 0.1% Triton X-100 (see Supplementary methods), overnight at 4 °C. Sections were then washed with 3 × 10 min PBS followed by a 1.5-h incubation in a mixture of Alexa Fluor^™^ secondary antibodies diluted in PBS-azide 0.1% at RT (see Supplementary methods). Sections were cover slipped with Dako fluorescence mounting media. Slides were examined and imaged using an LMS800 confocal microscope.

### Determination of total number of ganglia and neurons in the rat stomach

Total number of ganglia and neurons were determined in tissues from 4 female and 4 male rats that was cleared to allow visualisation of fluorescence in full thickness preparations using the clearing-enhanced 3D (Ce3D) clearing method (Bossolani et al. 2018; Li et al. 2019).

Rats were deeply anaesthetised with an intraperitoneal injection of a mixture of ketamine (55 mg/kg) and xylazine (9 mg/kg) prior to being perfused transcardially with heparinised PBS (5 U/mL heparin, Sigma-Aldrich) followed by fixative (2% paraformaldehyde and 0.2% picric acid, as above). The stomach was removed and post-fixed overnight at 4 °C in the same fixative, before being cleared with 3 × 10 min washes in DMSO, 3 × 10-min washes in PBS and then stored in PBS-azide at 4 °C. The stomachs were opened along the greater curvature and laid flat. The surface areas of the fundus, corpus and antrum were photographed and measured, and sample areas were removed and stained for human anti-Hu in wholemounts (see Supplementary methods). Full thickness preparations, the complete external muscle and mucosa, (~ 0.5 × 0.5 cm) from the fundus, corpus and antrum were removed and placed in a blocking buffer (PBS-azide with 0.3% Triton X-100, 1% bovine serum albumen (BSA), and 1% NHS) for 24 h at 37 °C before being incubated with primary antibody, human anti-Hu (Supplementary methods), diluted in the same blocking solution, for 4 days at 37 °C followed by 1 day at RT. Thickmounts were washed 3 × 30 min followed by 1 × overnight wash with a washing buffer (PBS-azide with 0.3% Triton X-100 and 0.5% 1-thioglycerol). They were then placed in Alexa Fluor^™^ secondary antibody (Supplementary methods) diluted in PBS-azide containing 0.3% Triton X-100 and incubated for 2 days at 37 °C. The tissues were washed again with a washing buffer, 3 × 30 min followed by 2 × overnight washes. The samples were then incubated in a Ce3D clearing solution (22% N-methylacetamide, 86% Histodenz, 0.1% Triton X-100, 0.5% 1-thioglycerol) for 3 days, which was changed with a fresh solution every 24 h. The wholemounts were mounted on standard microscope slides in the Ce3D clearing solution, and a coverslip was fixed on top, using a spacer to accommodate the thickness of the tissue. Full thickness z-series tile scan images through the myenteric plexus (MP) and submucosal plexus (SMP) were imaged with a LSM800 confocal microscope (Zeiss). Images were analysed using Zen software (Zeiss, Sydney, Australia) and ImageJ (https://imagej.nih.gov/ij/). In brief, using the 3D ImageJ suite and MorphoLibJ plugins (see Supplementary methods), a script was created to identify positive staining, above a threshold set by the experimenter, and segment it into 3 dimensional objects that were used to separate and outline individual ganglia in order to collect data for quantification. Separate ganglia were defined as having more than one cell width (20 µm) space between closest neurons. The 3-dimensional ganglion objects were projected into a single layer and converted to a 2-dimensional outline to measure the 2D size profiles of the ganglia. Ganglia were manually assigned as either MP or SMP as defined by their position in the z stack.

It is noted that several anaesthetic regimes were used. To reduce the use of animals, we have utilized animals for multiple purposes where possible. For this reason, we have taken tissues from animals that had a different anaesthesia.

## Results

### NADPHd histochemistry

**Fig. 1:**
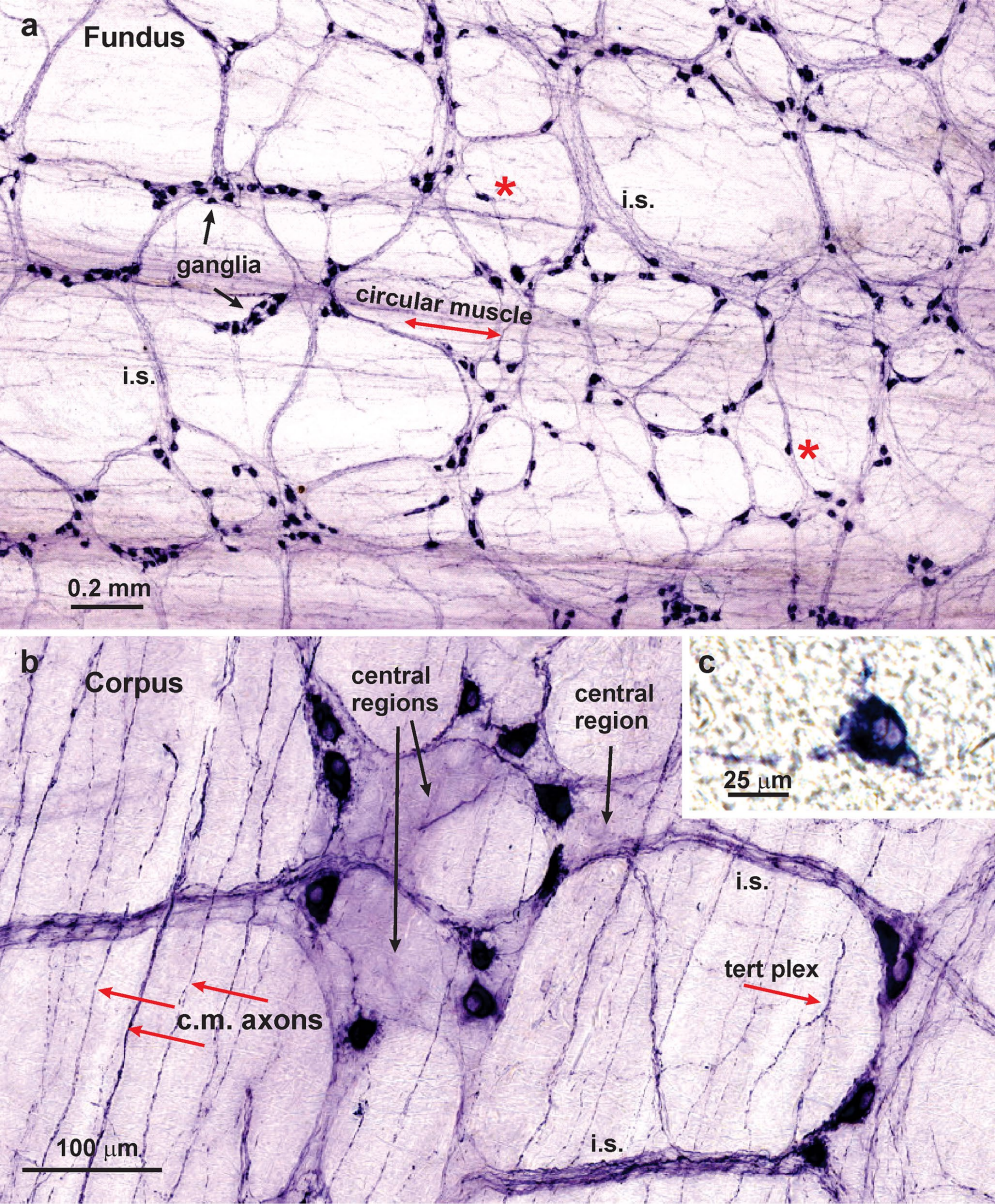
Neuronal NOS neurons revealed by NADPHd histochemistry in wholemounts of the rat stomach. (**a**): Fundus. Nerve cells were found in myenteric ganglia, in which they appeared to be randomly distributed, here and throughout the stomach. Some single nerve cells were also observed (*asterisks*). NADPHd was seen in fibres of internodal strands (*i.s.*) that connect the ganglia and in residual circular muscle. (**b**): Corpus. Myenteric ganglia viewed at higher power. It is apparent that there are no pericellular baskets of NADPHd positive nerve fibres around either the NADPHd positive or NADPHd negative nerve cells. Central regions of ganglia which contain unreactive nerve cells, but no perineuronal endings, are indicated. On the other hand, positively stained nerve fibres can be seen innervating the circular muscle (*c.m. axons*), in internodal strands (*i.s.*) and in the tertiary plexus (*tert plex*). (**c**): A submucosal neuron in the corpus revealed by NADPHd histochemistry. Typically, submucosal ganglia were very small and single NADPHd neurons were observed in the ganglia. Refractile connective tissue can be seen in the background

**Fig. 2:**
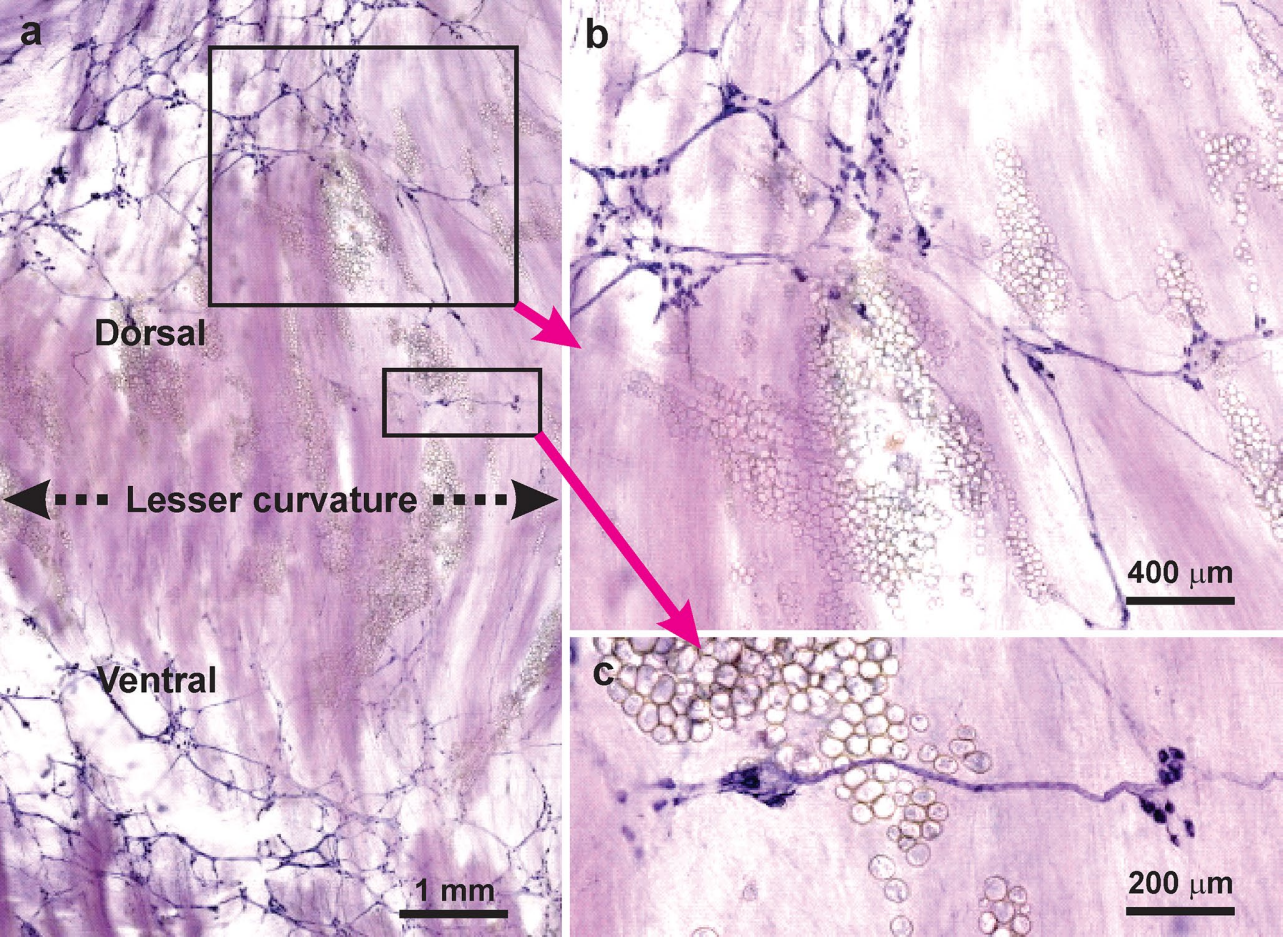
The appearance of the myenteric plexus of the lesser curvature stained for NADPHd histochemistry. (**a**): Along the lesser curvature and for 1.5–2 mm lateral to the lesser curvature on the adjacent dorsal and ventral gastric surfaces, there was an absence, or very few myenteric ganglia. (**b**): An area of panel a, indicated by the rectangle, shown at greater magnification. A region of ganglionated plexus is adjacent to regions largely free of ganglia. Small adherent fat deposits that are found along the attachment of the lesser omentum are seen. (**c**): Two small myenteric ganglia found close to the lesser curvature (enlargement of region in panel a)

The overall distribution of nNOS neurons was investigated using NADPHd histochemistry and immunohistochemistry for neuronal nNOS. NADPHd histochemistry revealed positive neurons in myenteric ganglia and allowed the organisation of the plexuses to be readily revealed (Fig. 1). Representative images are shown in this and in subsequent figures. NADPHd histochemistry showed myenteric ganglia and ganglionic connectives to be present throughout the stomach, except that there was a region along the lesser curvature that contained few ganglia (Fig. 2). The same paucity of ganglia at the lesser curvature was observed in preparations stained for neuronal NOS and the marker of the total neuron population, Hu. NADPHd positive neurons were also found in the much rarer submucosal ganglia (Fig. 1).

**Fig. 3:**
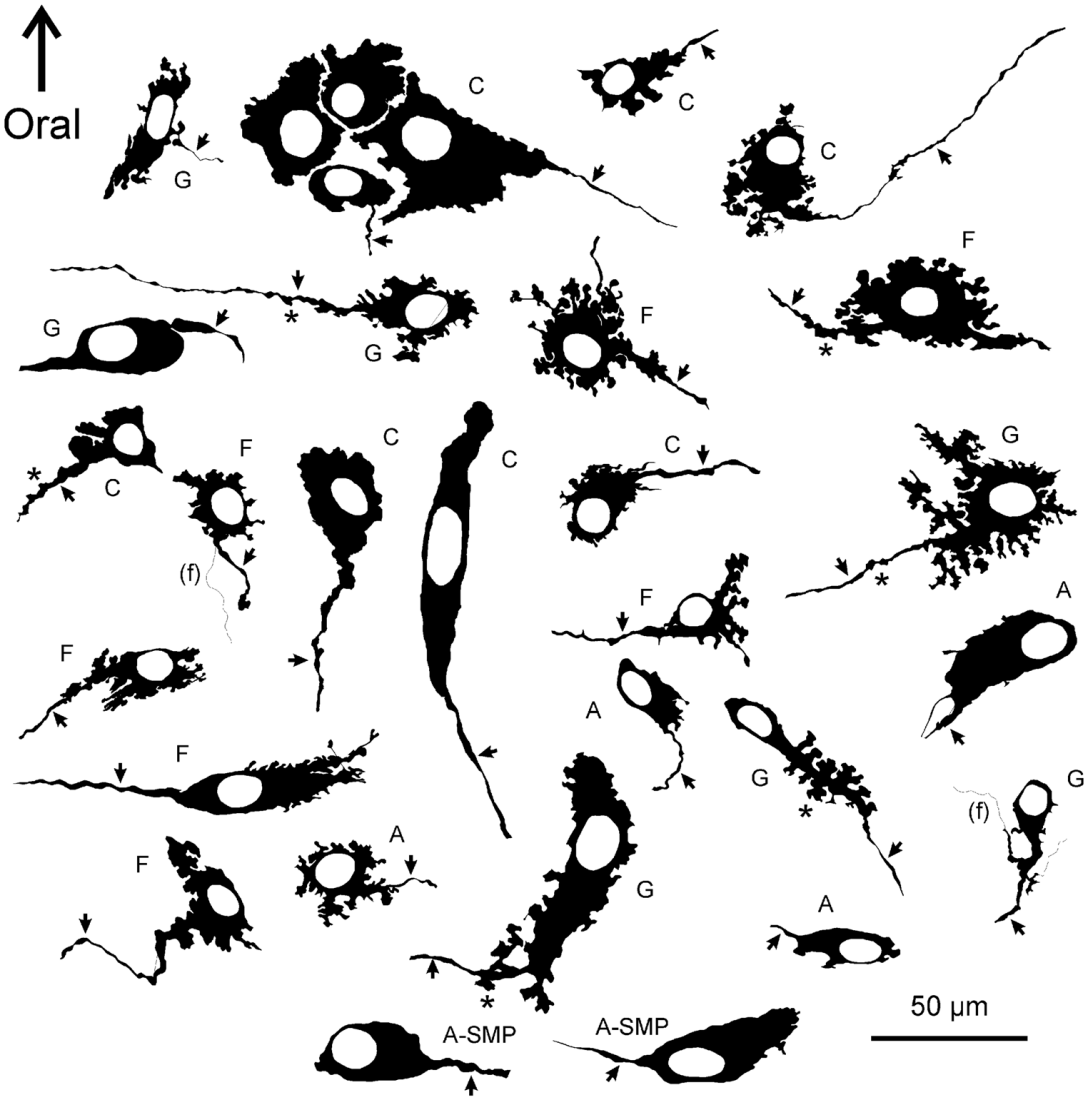
Camera lucida tracings of NADPHd positive nerve cells from different gastric regions. These images make it possible to see the fine details of nerve cell morphologies. From the myenteric plexus are neurons labelled A, antrum; C, corpus; F, fundus; and G, the gradient region in which the mucosa of the corpus and antrum merge. Neurons labelled A-SMP are from submucosal ganglia of the antrum. The neurons have single axons (*arrows*) and commonly have elaborate lamellar dendrites. Examples of axonal spines (*asterisks*) are indicated. Some fine fibres that cross cell bodies are included (*f*). There was a wide range of sizes (see also Fig. 5e)

Finer details of cell body morphology, and specialisations such as axonal spines, were revealed using camera lucida imaging (Fig. 3). The cell bodies had a wide size range (Figs. 1 and 3). In many cases the axons of reactive neurons could be traced from the cell bodies. The great majority of neurons had only one identifiable axon that sometimes exhibited axonal spines (Fig. 3). In addition, short lamellar dendrites arising from the cell bodies were seen (Fig. 3). The presence of a single axon and lamellar dendrites identifies these as type I neurons in the classification of Dogiel (Dogiel 1899; Brehmer et al. 1999; Furness 2006). Fine varicose fibres, that are described in detail later, were found in the muscle layers. However, neither NADPHd histochemistry or nNOS immunohistochemistry stained pericellular baskets of nerve fibres that are typically seen for fibres with other chemistries in myenteric ganglia (Fig. 1).

### Relation of NADPH diaphorase and neuronal NOS staining

**Fig. 4:**
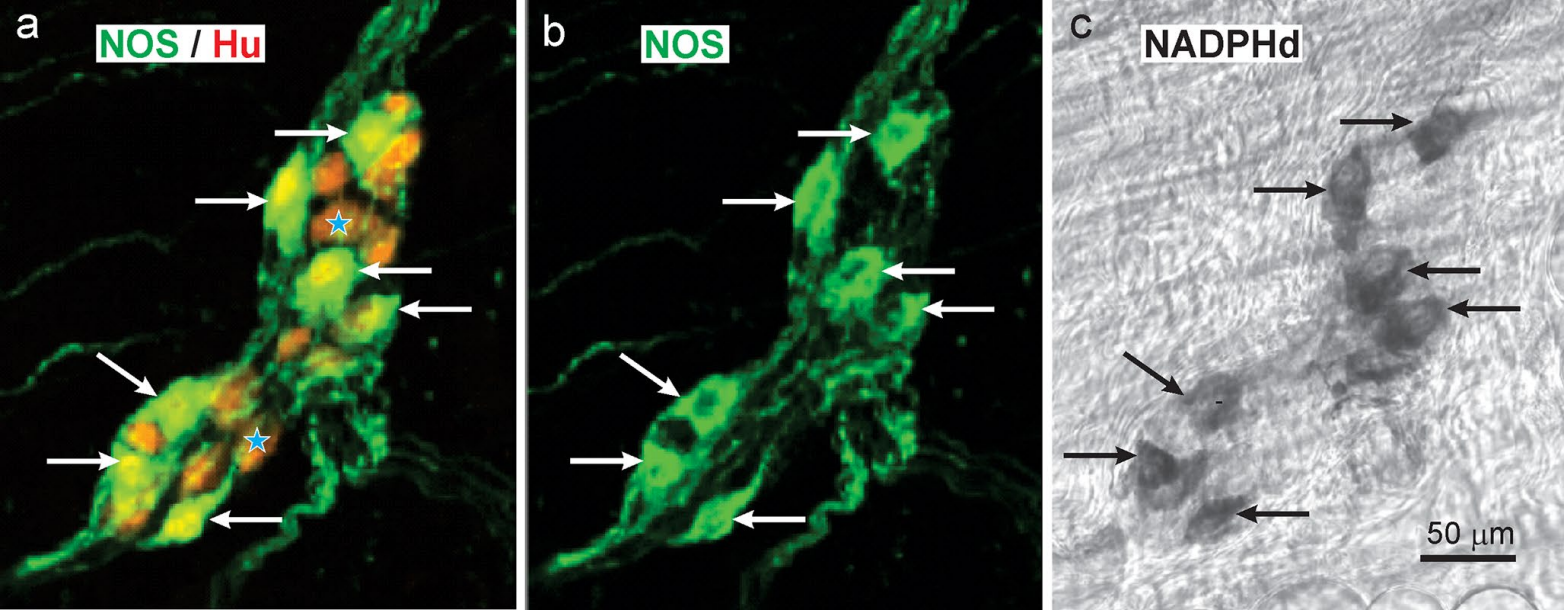
Localisation of nNOS immunoreactivity (**b**) and NADPH diaphorase activity (**c**) in the same neurons of a myenteric ganglion in the rat antrum. Wholemounts were first incubated with a mixture of anti-nNOS (green) and anti-Hu (red) antibodies and processed for immunohistochemistry. Tissue was mounted on microscope slides and selected ganglia were photographed (**a**, **b**). The wholemounts were then removed from the slides, washed, and reacted for NADPH diaphorase histochemistry (**c**). Neurons with nNOS immunoreactivity were also NADPHd positive and vice-versa (arrows). There were slight changes of relative neuron positions caused by unmounting and remounting the tissue

NADPH diaphorase is the colour reaction that reveals the presence of NADPH oxidases, and because nNOS is a NADPH oxidase, it is revealed by NADPHd histochemistry. However, many other enzymes use NADPH as a substrate, meaning that the NADPHd reaction is not unique to NOS. Therefore, we reacted gastric samples successively for nNOS immunohistochemistry and NADPHd. Preparations stained for neuronal NOS and Hu that were restained for NADPHd histochemistry showed that the same neurons were revealed by both methods (Fig. 4). There were slight positional shifts between images taken of the fluorescence versus the NADPHd because the wholemounts had to be removed from the microscope slides and the mounting medium washed out before applying the NADPHd method and re-mounting the specimens (Fig. 4).

### Relation of neurons with nNOS immunohistochemistry to the overall population

**Fig. 5:**
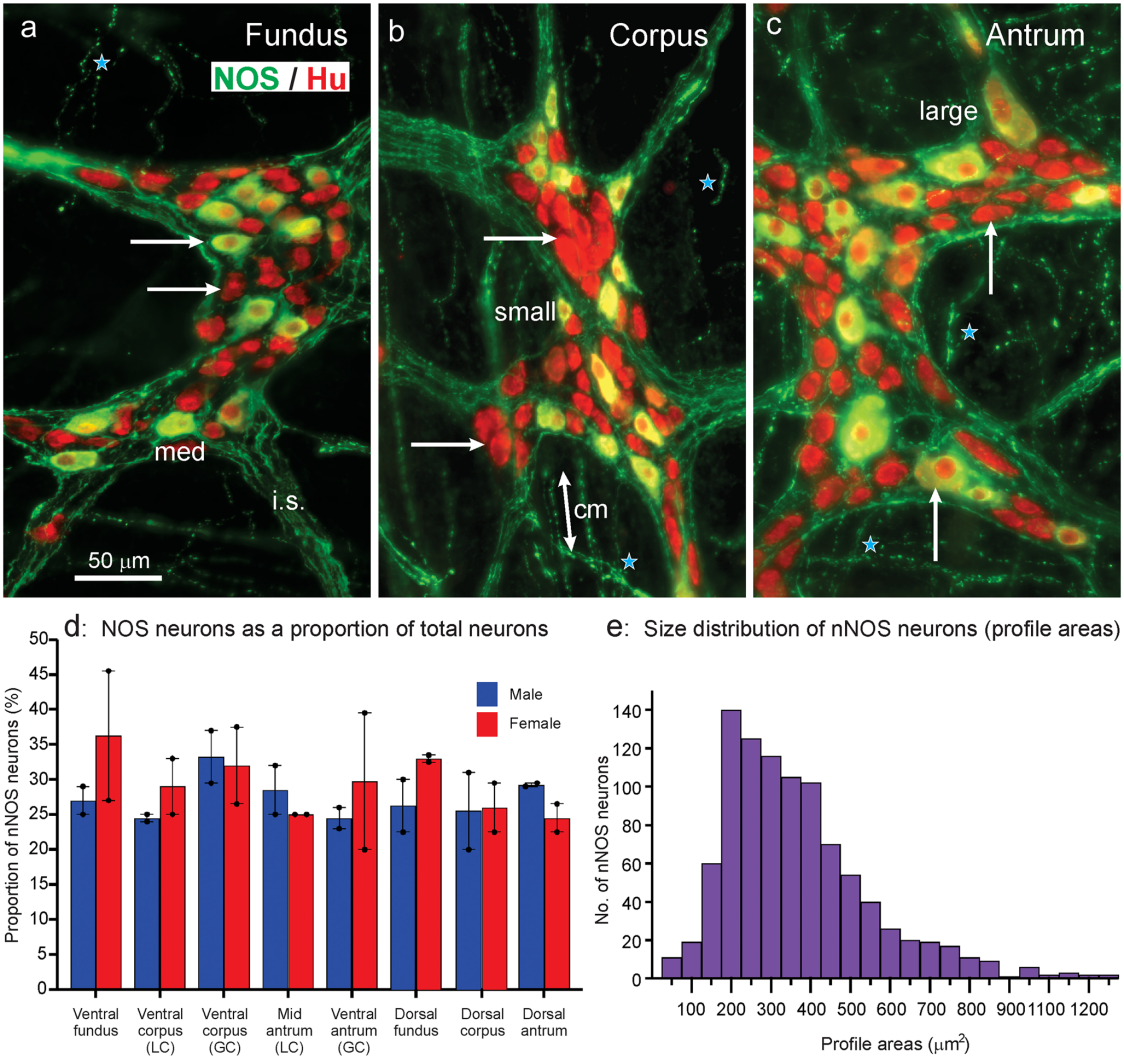
Colocalisation of immunoreactivity for nNOS and Hu in myenteric ganglia of the fundus (**a**), corpus (**b**) and antrum (**c**). Hu immunoreactivity (red) can be seen in the nuclei of nNOS neurons, whose cytoplasms appear yellow/green. Hu is red in the nuclei and cytoplasm of nNOS negative neurons. The nNOS cell bodies are scattered in what appears to be a random fashion in the ganglia. Neuronal NOS is in nerve fibres in the internodal strands (*i.s.*) that connect ganglia, in fibres in the circular muscle (*cm*) and in the tertiary plexus (*stars*). Nerve cell bodies, examples of which are indicated by arrows, do not have pericellular nNOS fibre baskets. There is a wide range of sizes of nNOS neuron cell bodies; examples of small, medium (*med*) and large neurons are indicated. The micrographs are maximum intensity projections from z-series images. (**d**): Percentages of Hu immunoreactive nerve cell bodies that had nNOS immunoreactivity in the myenteric plexus of different gastric regions (see Supplementary methods) and in male and female rats (each *n* = 4). GC, greater curvature; LC, lesser curvature. The ventral antrum includes a transition (gradient) between corpus and antrum. (**e**): Profile areas of nNOS immunoreactive neurons measured from myenteric ganglia visualised in wholemounts. Each bar is a 50-µm interval, mid values indicated for every second bar

Neuronal NOS immunoreactive cell bodies were scattered in myenteric ganglia, not forming clumps or having any preferred location (Fig. 5). Similar proportions of myenteric neurons had nNOS immunoreactivity throughout the stomach, and no significant differences were seen between female and male rats (Fig. 5d). The average proportion of nerve cells that were nNOS immunoreactive in the whole stomach was 28.9 ± 1.0% (*n* = 4, 8 regions from 4 rats; 6400 Hu positive cells counted). The profile areas of the nerve cells were determined using Zen Blue software (see Supplementary methods) for a total of 1857 nNOS positive cells from the 8 regions of the 4 stomachs. Almost all the cells included in the cell size analysis were both nNOS positive and Hu positive. A rare population of small-sized nNOS positive and Hu negative cells (0.9% of nNOS neurons) were included in the cell size analysis. Neurons with nNOS immunoreactivity were also scattered in the much rarer submucosal ganglia.

### Locations of nNOS nerve terminals

**Fig. 6:**
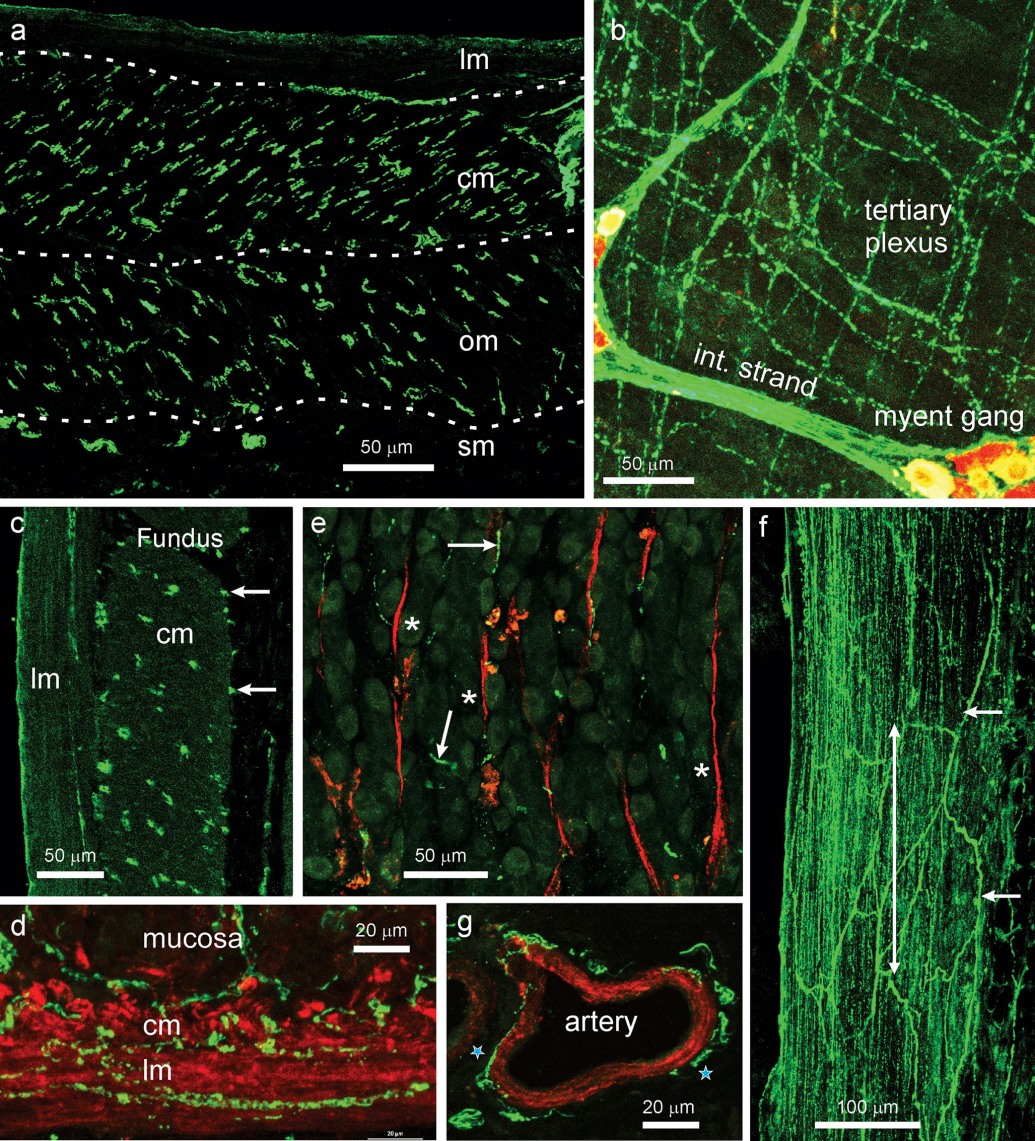
Distributions of nNOS nerve terminals in the rat stomach; nNOS in green, smooth muscle in red (**e**, **d**, **g**), Hu in red (**b**). (**a**): Section from the corpus external muscle, lateral to the esophago-gastric junction, shows terminals throughout the thickness of the circular (*cm*) and oblique muscle (*om*). Very rare fibres were present in the longitudinal muscle (*lm*). The dashed lines mark the boundaries of the layers. Some fibre bundles are in the submucosa (*sm*). (**b**): In wholemounts, nNOS nerve terminals were found in the tertiary plexus. This plexus is believed to innervate the longitudinal muscle. An internodal strand (*int strand*) and a ganglion of the myenteric plexus (*myent gang*) are in the same plane as the tertiary plexus (Hu positive neurons are red). (**c**). External muscle of the fundus. nNOS fibres are seen throughout the thickness of the circular (*cm*), but not the longitudinal muscle (*lm*). Some fibres are adjacent to the inner surface of the circular muscle (arrows). (**d**): nNOS fibres are related to both the circular (*cm*) and longitudinal muscle (*lm*) layers of the muscularis mucosae (image from antrum). (**e**): Smooth muscle strands (actin, red) in the mucosa of the corpus (*asterisks*). These seldom had close approaches of nNOS fibres (*arrows*). (**f**): nNOS innervation of the esophago-pyloric ligament. Nerve fibres within the ligament run parallel to its length (*double headed arrow*). A network of small fibre bundles is at the ligament surface (*arrows*). (**g**): nNOS immunoreactive fibres around a small artery within the gastric wall. Fibresclose to the arterial muscle are indicated by the *stars*

Two targets of nNOS neurons, gastric muscle and small arteries within the wall of the stomach, were identified (Fig. 6). In sections taken close to the esophago-gastric junction, the three external muscle layers could be seen (Fig. 6a). Innervation by nNOS immunoreactive fibres was observed throughout the thickness of both the circular and oblique muscles. Fibres providing a regular innervation within the longitudinal muscle did not occur, although single fibres were occasionally found within this layer.

In other regions of the gastrointestinal tract, where the longitudinal muscle is thinner than about 50 µm, it is innervated by fibres at its inner surface that form the tertiary component of the myenteric plexus, commonly known as the tertiary plexus (Llewellyn-Smith et al. 1993; Furness 2006). Consistent with this, nNOS immunoreactive nerve fibres were prominent in the tertiary plexus of the stomach (Fig. 6b). In larger mammals, where the muscle is thicker, the innervation is throughout the layer. Some of the fibres innervating the circular muscle were adjacent to its inner surface. This was apparent in regions where there was no internal oblique muscle (Fig. 6c). Neuronal NOS fibres also supplied the muscularis mucosae, and could be seen associated with both its layers (Fig. 6d). Strands of smooth muscle also occur between mucosal glands in the corpus and antrum, but these very rarely had nNOS fibres adjacent to them (Fig. 6e). A small number of fine nNOS immunoreactive fibres was seen in most mucosal sections, but they seldom appeared to follow the muscle strands. The esophago-pyloric ligament was innervated throughout its thickness by varicose nNOS immunoreactive fibres, most of which ran parallel to the ligament (Fig. 6f). Arteries throughout the stomach wall were consistently innervated by perivascular networks of nNOS fibres close to the arterial muscle (Fig. 6g).

**Fig. 7:**
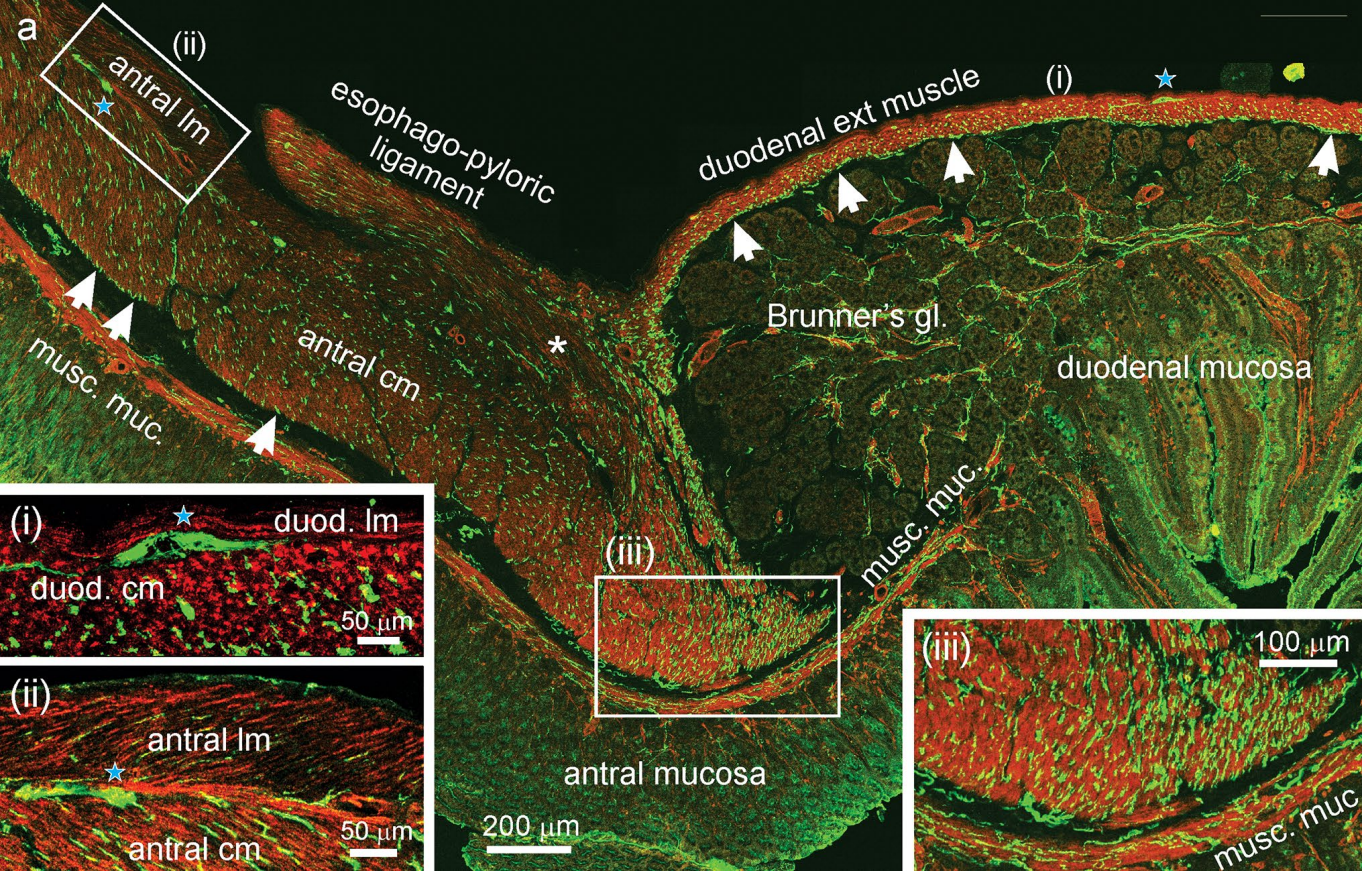
Distributions of nNOS terminals at the gastro-duodenal junction. In all the images, nNOS immunoreactivity is in green, smooth muscle actin immunoreactivity is in red. (**a**) (Main panel): low power view of the junction. The pyloric end of the esophago-pyloric ligament and its insertion at the pyloric sphincter region (*asterisk*) can be seen. The antral muscle thickens slightly to form the pyloric sphincter following which is a rapid transition from the thick antral muscle to the thin external muscle of the duodenum. The duodenum immediately adjacent to the pylorus is dominated by the submucosal glands of Brunner (*Brunner’s gl.*). There is a prominent network of nNOS fibres at the internal surface of the circular muscle of the duodenum (arrows in (**a**), at right). This is the deep muscular plexus. nNOS positive myenteric neurons can be seen in the layers between the longitudinal and circular muscle (*blue stars*). The longitudinal muscle was only about 10-µm thick in the duodenum. Neuronal NOS fibres (*arrowed*) are also seen at the inner surface of the antral circular muscle (arrows in a, at left). There were very few nNOS immunoreactive axons in the antral and duodenal longitudinal muscle (insets, **i**, **ii**). The antral (pyloric sphincter) muscle adjacent to the duodenum is densely innervated (inset, **iii**). Fibres immunoreactive for nNOS were rare in the mucosa

Innervation of the pyloric sphincter by nitrergic inhibitory neurons is important to regulate passage of gastric content to the duodenum (Mashimo 1996). The sections through the sphincter region showed a slight thickening of the circular muscle, which was more densely innervated than the antral circular muscle further from the sphincter region (Fig. 7).

### Number of ganglia and neurons

**Fig. 8:**
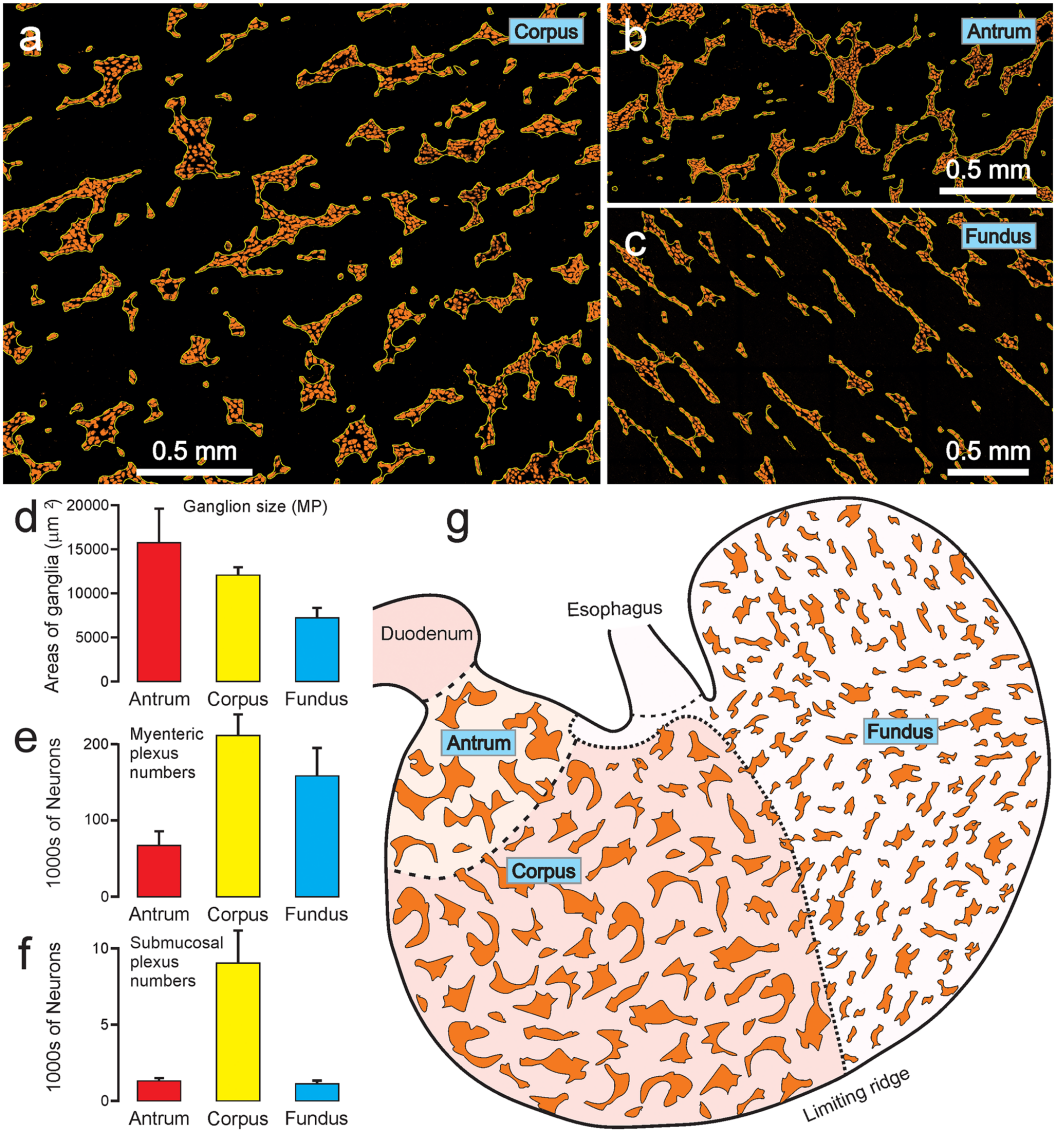
Sizes and distributions of rat gastric ganglia. (**a**), (**b**) and (**c**) show images of the myenteric plexus in the corpus, antrum and fundus, from wholemounts stained for nNOS. (**d**): Average areas of myenteric ganglia of the three regions. Ganglia were largest in the antrum. (**e**, **f**): Total numbers of myenteric (**e**) and submucosal (**f**) neurons in the regions. (**g**): Representation of the rat stomach to illustrate the distributions and relative sizes of myenteric ganglia

To determine the density of occurrence of myenteric and submucosal ganglia, and total neuron number, stomachs were opened out along the greater curvature and laid flat, which required some cuts to be made (see Supplementary methods). The surface areas of the fundus, corpus and antrum were measured, and sample areas were removed and stained for Hu in thickmounts (Fig. 8). The ganglia were outlined and counted, and their areas were measured. The number of neurons per region was calculated from the number of ganglia per area and neurons per ganglion (see Supplementary methods; Fig. 8; Table 1). Myenteric ganglia in the antrum were about 1.3 times the size of those in the corpus, which in turn were about 2.3 times the size of those in the fundus (Fig. 8d). In terms of number of myenteric neurons per region, the antrum contained 67,031 ± 19,410, the corpus 211,778 ± 27,252 and the fundus 158,781 ± 36,908. The total number of myenteric neurons was 437,591 ± 49,816, rounded to 438,000 ± 50,000 (mean ± SEM), that is, our data indicates a total gastric myenteric neuron population between about 388,000 and 488,000 neurons. The number of submucosal neurons, rounded, was about 11,400 +/- 2200 neurons (Table 1; Fig. 8f), which is about 2.6% of the number in myenteric ganglia.

**Table 1:** Average data concerning enteric ganglia and nerve cells in the myenteric and submucosal ganglia in each region of the rat stomach (*n* = 8, 4 male, 4 female rats, 6.5 to 8 weeks old)

Myenteric ganglia							
	Ganglia/mm^2^	Ganglion size (µm^2^)	Neurons/ganglion	Neurons/mm^2^	Area of region (mm^2^)	Neurons/region	SEM
Antrum	9.9 ± 0.7	15,723 ± 3899	42.7 ± 10.7	423.6 ± 115.1	158.2 ± 15.5	67,031	19,410
Corpus	10.1 ± 0.7	12,080 ± 945	26.0 ± 2.3	263.3 ± 29.9	804.3 ± 48.6	211,779	27,252
Fundus	15. 4 ± 1.6	5250 ± 810	13.5 ± 2.0	208.5 ± 42.5	761.6 ± 81.9	158,781	36,908
					**Total neurons**	**437,591**	**49,816**
					**Rounded**	**438,000**	**50,000**
**Submucosal ganglia**							
	Ganglia/mm^2^	Ganglion size (µm^2^)	Neurons/ganglion	Neurons/mm^2^	Area of region (mm^2^)	Neurons/region	SEM
Antrum	4.7 ± 0.5	662 ± 31	1.7 ± 0.1	8.1 ± 1.7	158.2 ± 15.5	1286	216
Corpus	4.9 ± 1.0	989 ± 67	2.3 ± 0.2	11.2 ± 2.6	804.3 ± 48.6	9021	2144
Fundus	1.0 ± 0.2	680 ± 46	1.5 ± 0.2	1.4 ± 0.3	761.6 ± 81.9	1096	252
					**Total neurons**	**11,403**	**2169**
					**Rounded**	**11,400**	**2200**

## Discussion

This study shows that nitrergic neurons in the rat stomach supply the longitudinal, circular and oblique layers of the external muscle, the muscularis mucosae and arteries within the gastric wall. A small number of fibres were in the mucosa. All the neurons appeared to have a single axon and in most cases a type I morphology, a shape originally described by Dogiel (1899). Type I is the typical morphology of enteric motor neurons that supply the muscle of the gastrointestinal tract (Costa et al. 1996; Brookes et al. 1998). However, some nNOS immunoreactive interneurons that supply varicosities surrounding myenteric neurons in the small and large intestines, also have type I morphology (Costa et al. 1992; McConalogue and Furness 1993). By contrast, in the present study of the rat stomach, we found that nNOS neurons did not supply terminals around myenteric nerve cells, whether NADPHd histochemistry or nNOS immunohistochemistry was used to locate the terminals of nNOS neurons. Thus, nNOS appears to be exclusively in motor neurons supplying the muscle of the rat stomach. This may be different from other species. In the guinea-pig stomach, some myenteric neurons of the gastric corpus, that had been labelled by intracellular dye injection and subsequently labelled for NADPHd, had axons that projected to myenteric ganglia (Schemann and Schaaf 1995). The authors concluded that 57% of nNOS neurons were motor neurons, and 39% were non-motor, some of which projected to other myenteric ganglia. Although, from consideration of their targets, we conclude that nNOS immunoreactivity identifies 4 classes of motor neurons that supply the muscle of the rat stomach, an analysis of cell body sizes (Fig. 5) did not identify separate grouping of neurons. Thus, if the classes of gastric motor neurons have different sizes, as might be suggested from comparisons of small motor neurons to the longitudinal muscle and larger motor neurons to the circular muscle in the small intestine (Brookes 2001), they must overlap in their size ranges to a sufficient extent to present an aggregated unimodal distribution (Fig. 5). In the guinea-pig, the inhibitory neurons supply the small regions of the gastric muscle, the majority of nerve cells projecting to the muscle 2 mm or less anal and 5 mm or less in the circumferential direction (Brookes et al. 1998). The large number of neurons and their local projections to targets suggests that there is a finely graded control of motor functions in the stomach by the recruitment of different numbers of motor neurons. This presumed grading has not been closely investigated functionally. We also found nNOS fibres around the arteries in the stomach wall. We presume that these may be axons of enteric vasodilator neurons (Furness et al. 2020), but we cannot exclude an extrinsic origin of these nNOS fibres.

In the current study, we found similar proportions of myenteric neurons were nNOS immunoreactive throughout the rat stomach, with an average of 29% of neurons being nNOS across all the regions. This is the same as counts in the guinea-pig corpus which showed that 29% of nerve cells in the myenteric plexus were immunoreactive for nNOS (Schemann et al. 1995). In the human stomach, 27% of myenteric neurons are nNOS immunoreactive (Anetsberger et al. 2018). Overall, the number of nNOS neurons in myenteric ganglia of the rat, calculated from the total neuron counts (Table 1), was estimated to be 19,000 in the antrum, 61,000 in the corpus and 46,000 in the fundus. These large numbers reinforce the hypothesis that there may be a close grading of control through the recruitment of different numbers of neurons. It would also be consistent with there being some redundancy of neuron numbers.

It was notable that nNOS nerve fibres were uncommon within the longitudinal muscle compared to the dense innervation of the circular and oblique muscle layers (e.g., Figs. 6 and 7). In addition, we found a region of very dense nNOS fibre innervation of the gastric circular muscle close to the junction with the duodenum. Few fibres within the longitudinal and many in the circular muscle parallel innervation patterns seen in other gastrointestinal regions. In parts of the gut where the longitudinal muscle is thin (less than about 50 µm), it is innervated by a plexus of nerve fibres at its inner surface, the tertiary plexus, not by fibres within the layer (Llewellyn-Smith et al. 1993; Furness 2006). A tertiary plexus was apparent in wholemounts of the myenteric plexus and longitudinal muscle of the rat stomach (Fig. 6b). In dog and human stomach in which muscle layers are thicker than rat, inhibitory nerve fibres identified by either nNOS or VIP immunoreactivity are found throughout the thickness of the longitudinal muscle (Furness et al. 1991; Smith et al. 2001). The tertiary plexus is also prominent in the guinea-pig stomach, but in this species there are also inhibitory nerve fibres, revealed by VIP immunoreactivity, within the longitudinal muscle layer (Mawe et al. 1989).

*In conclusion*, the rat stomach harbours large numbers of nNOS neurons, all or almost all of which are motor neurons supplying the external muscle, the muscularis mucosae and intramural arteries.

## Supplementary Information

Below is the link to the electronic supplementary material.

Supplementary file1 (DOCX 502 KB)

## References

[CR1] Anetsberger D, Kürten S, Jabari S, Brehmer A (2018). Morphological and immunohistochemical characterization of human intrinsic gastric neurons. Cells Tissues Organs.

[CR2] Boeckxstaens GE, Pelckmans PA, Bogers JJ, Bult H, De Man JG, Oosterbosch L, Herman AG, Van Maercke YM (1991). Release of nitric oxide upon stimulation of nonadrenergic noncholinergic nerves in the rat gastric fundus. J Pharmacol Exp Ther.

[CR3] Boeckxstaens GE, Pelckmans PA, De Man JG, Bult H, Herman AG, Van Maercke YM (1992). Evidence for a differential release of nitric oxide and vasoactive intestinal polypeptide by noradrenic noncholinergic nerves in the rat gastric fundus. Arch Int Pharmacodyn Ther.

[CR4] Bossolani GDP, Pintelon I, Detrez JD, Buckinx R, Thys S, Zanoni JN, De Vos WH, Timmermans J-P (2018) Comparative analysis reveals Ce3D as optimal clearing method for in toto imaging of the mouse intestine. Neurogastroenterol Motil 31:e13560 10.1111/nmo.13560 30761698

[CR5] Brehmer A, Schrödl F, Neuhuber W (1999). Morphological classifications of enteric neurons- 100 years after Dogiel. Anat Embryol.

[CR6] Brookes SJH (2001). Classes of enteric nerve cells in the guinea-pig small intestine. Anat Rec.

[CR7] Brookes SJH, Hennig G, Schemann M (1998). Identification of motor neurons to the circular muscle of the guinea pig gastric corpus. J Comp Neurol.

[CR8] Costa M, Brookes SJH, Steele PA, Gibbins I, Burcher E, Kandiah CJ (1996). Neurochemical classification of myenteric neurons in the guinea-pig ileum. Neuroscience.

[CR9] Costa M, Furness JB, Pompolo S, Brookes SJH, Bornstein JC, Bredt DS, Snyder SH (1992). Projections and chemical coding of neurons with immunoreactivity for nitric oxide synthase in the guinea-pig small intestine. Neurosci Lett.

[CR10] Desai KM, Sessa WC, Vane JR (1991). Involvement of nitric oxide in the reflex relaxation of the stomach to accommodate food or fluid. Nature.

[CR11] Desai KM, Warner TD, Bishop AE, Polak JM, Vane JR (1994). Nitric oxide, and not vasoactive intestinal peptide, as the main neurotransmitter of vagally induced relaxation of the guinea pig stomach. Br J Pharmacol.

[CR12] Dogiel AS (1899). Über den Bau der Ganglien in den Geflechten des Darmes und der Gallenblase des Menschen und der Säugetiere. Arch Anat Physiol Leipzig Anat Abt Jg.

[CR13] Fahrenkrug J, Haglund U, Jodal M, Lundgren O, Olbe L, Schaffalitzky de Muckadell O (1978). Nervous release of vasoactive intestinal polypeptide in the gastrointestinal tract of cats: possible physiological implications. J Physiol (lond).

[CR14] Furness JB (2006). The Enteric Nervous System.

[CR15] Furness JB, Lloyd KCK, Sternini C, Walsh JH (1991). Evidence that myenteric neurons of the gastric corpus project to both the mucosa and the external muscle: myectomy operations on the canine stomach. Cell Tissue Res.

[CR16] Furness JB, Di Natale M, Hunne B, Oparija L, Ward SM, Sasse KC, Powley TL, Stebbing MJ, Jaffey J, Fothergill LJ (2020). The identification of neuronal control pathways supplying effector tissues in the stomach. Cell Tissue Res.

[CR17] Gil V, Martínez-Cutillas M, Mañé N, Martín MT, Jiménez M, Gallego D (2013). P2Y_1_ knockout mice lack purinergic neuromuscular transmission in the antrum and cecum. Neurogastroenterol Motil.

[CR18] Huang PL, Dawson TM, Bredt DS, Snyder SH, Fishman MC (1993). Targeted disruption of the neuronal nitric oxide synthase gene. Cell.

[CR19] Idrizaj E, Traini C, Vannucchi MG, Baccari MC (2021). Nitric oxide: from gastric motility to gastric dysmotility. Int J Mol Sci.

[CR20] Knowles RG, Moncada S (1994). Nitric oxide synthases in mammals. Biochem J.

[CR21] Li CG, Rand MJ (1990). Nitric oxide and vasoactive intestinal polypeptide mediate non-adrenergic, non-cholinergic inhibitory transmission to smooth muscle of the rat gastric fundus. Eur J Pharmacol.

[CR22] Li W, Germain RN, Gerner MY (2019). High-dimensional cell-level analysis of tissues with Ce3D multiplex volume imaging. Nat Protoc.

[CR23] Li ZS, Furness JB (2000). Inputs from intrinsic sensory neurons to NOS immunoreactive neurons in the myenteric plexus of guinea-pig ileum. Cell Tissue Res.

[CR24] Llewellyn-Smith IJ, Costa M, Furness JB, Bornstein JC (1993). Structure of the tertiary component of the myenteric plexus in the guinea-pig small intestine. Cell Tissue Res.

[CR25] Martinson J (1965). Vagal relaxation of the stomach. Experimental re-investigation of the concept of the transmission mechanism. Acta Physiol Scand.

[CR26] Mashimo H, He XD, Huang PL, Fishman MC, Goyal RK (1996) Neuronal constitutive nitric oxide synthase is involved in murine enteric inhibitory neurotransmission. J Clin Investig 98(1):8–13. 10.1172/JCI118781 10.1172/JCI118781 PMC507393 8690808

[CR27] Mawe GM, Schemann M, Wood JD, Gershon MD (1989). Immunocyctochemical analysis of potential neurotransmitters present in the myenteric plexus and muscular layers of the corpus of the guinea pig stomach. Anat Rec.

[CR28] McConalogue K, Furness JB (1993). Projections of nitric oxide synthesizing neurons in the guinea-pig colon. Cell Tissue Res.

[CR29] Michel K, Reiche D, Schemann M (2000). Projections and neurochemical coding of motor neurones to the circular and longitudinal muscle of the guinea pig gastric corpus. Pflügers Arch Eur J Physiol.

[CR30] Pfannkuche H, Reiche D, Sann H, Schemann M (1998). Different subpopulations of cholinergic and nitrergic myenteric neurones project to mucosa and circular muscle of the guinea-pig gastric fundus. Cell Tissue Res.

[CR31] Schemann M, Reiche D, Michel K (2001). Enteric pathways in the stomach. Anat Rec.

[CR32] Schemann M, Schaaf C (1995). Differential projection of cholinergic and nitroxidergic neurons in the myenteric plexus of guinea pig stomach. Am J Physiol.

[CR33] Schemann M, Schaaf C, Mäder M (1995). Neurotransmitter coding of enteric neurones in the guinea pig stomach. J Comp Neurol.

[CR34] Smith VC, Dhatt N, Buchan AMJ (2001). The innervation of the human antro-pyloric region: organization and composition. Can J Physiol Pharmacol.

[CR35] Young HM, Furness JB, Shuttleworth CWR, Bredt DS, Snyder SH (1992). Co-localization of nitric oxide synthase immunoreactivity and NADPH diaphorase staining in neurons of the guinea-pig intestine. Histochemistry.

