# Supplementary material for: Morphologies, dimensions and targets of gastric nitric oxide synthase neurons
Source: Cell Tissue Res. 2022 Feb 11;388(1):19–32. doi: 10.1007/s00441-022-03594-0 (PMC8976817; doi:10.1007/s00441-022-03594-0)
Supplement: Supplementary file 1 — Supplementary file1 (DOCX 502 KB) [file 441_2022_3594_MOESM1_ESM.docx]

**Supplementary Methods**

***Detection and quantitation of nNOS immunoreactive neurons***

To determine the proportions of neurons that were immunoreactive for nNOS, the neurons were counted from 8 regions (see diagram). These regions were the ventral fundus (1), ventral corpus closer to the lesser curvature (2), ventral corpus closer to the greater curvature (3), ventral antrum at the greater curvature, which includes the region of transition from corpus to antrum, (4), antrum closer to the lesser curvature (5), dorsal corpus (6), dorsal fundus (7), and dorsal antrum (8). From each region, 200 Hu positive cells were located, without knowledge of their being nNOS positive. Of the Hu positive cells, the number of nNOS positive cells was counted to obtain the proportions of nNOS neurons in that region. Counts were made in all 8 regions, across 4 rat stomachs (2 male, 2 female). The proportions of nNOS neurons in the stomach were calculated from 6400 Hu positive cells in total.

Cell sizes of nNOS positive cells were determined using Zen Blue software (Zeiss, Sydney, Australia). The Draw-Spline Contour tool in the Graphics menu was used to trace a border around the edges of nNOS positive cells, including the cell body and dendrites. The area (µm^2^) enclosed by the border was calculated by Zen measurement tools, this area represented the size of the cell profile. Cell sizes were determined from a total of 1857 nNOS positive cells from 8 regions from 4 stomachs (2 male, 2 female).


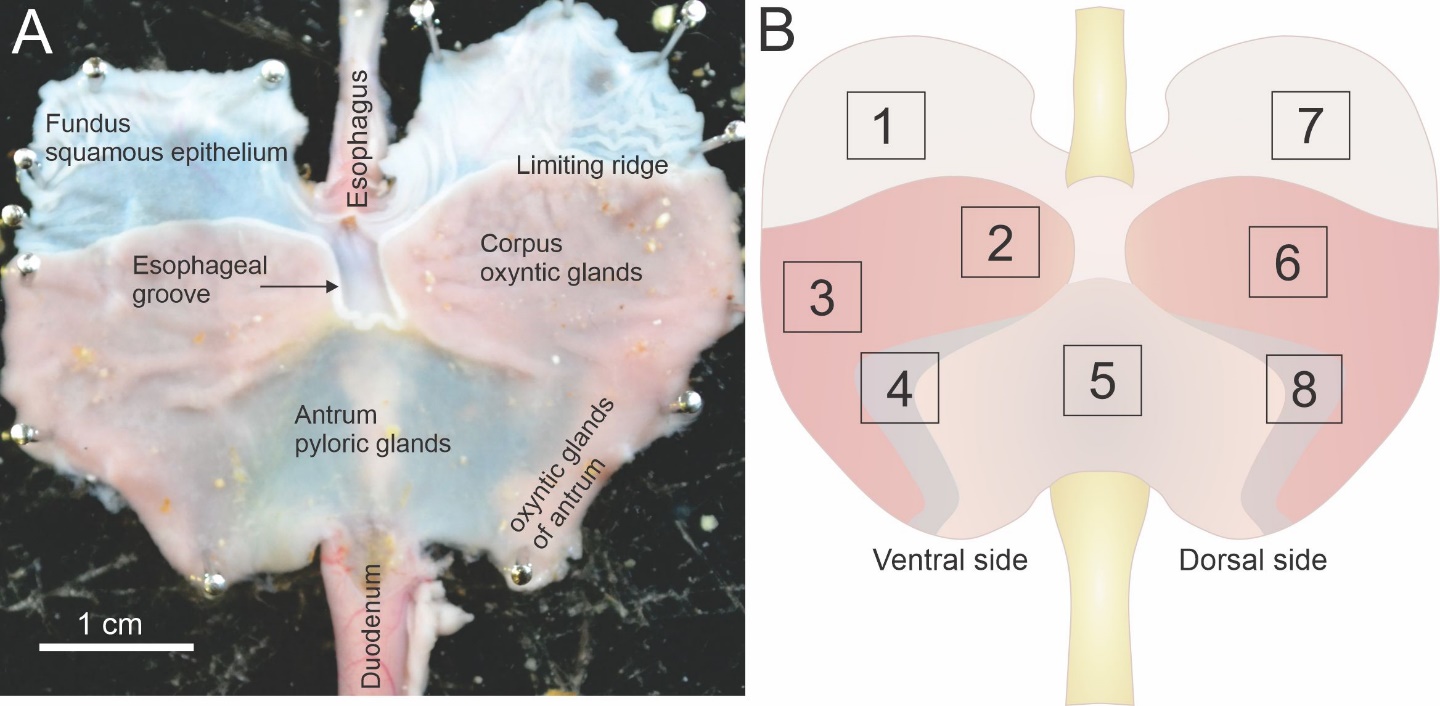


**Supplementary Fig. 1**. **A**. A fresh stomach opened along the greater curvature and stretched flat to show the luminal surface. **B**: A diagrammatical representation of the luminal surface of the stomach, the numbered boxes (see list above) indicating the regions of the stomach that were used to analyse the distribution of nNOS immunoreactive neurons. Regions 4 and 8 include the part of the mucosa that has a gradient of parietal cell occurrence, mucosal height, muscle thickness and gastrin cell occurrence between the corpus and antrum mucosa.

***Determination of total numbers of ganglia and neurons***

Stomachs from 6 female and 6 male rats were used. Fixed stomachs (see manuscript) were opened out and sample regions were cut from the tissue (Supplementary Fig. 2) and processed as described in the methods section of the paper.


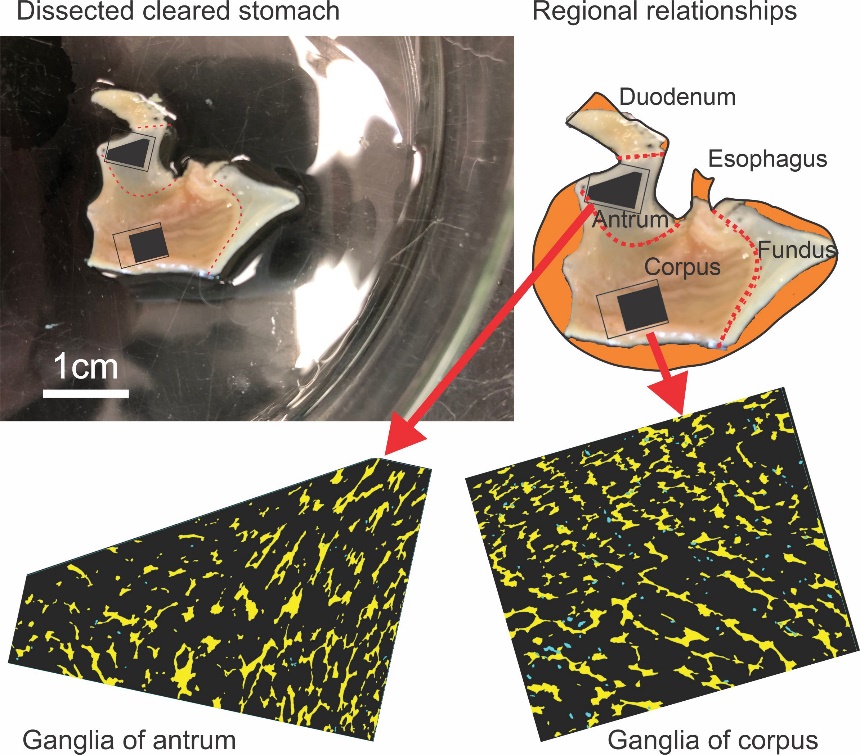


**Supplementary Fig. 2**. Tissue regions taken for clearing from a fixed stomach (not cleared yet), and a scaled representation of the ganglia revealed in each region by Hu staining after clearing. Myenteric ganglia have been coloured yellow, submucosal ganglia have been coloured blue. Part of the fundus had been removed). The stomach wall was pinned in a glass dish lined with silicone elastomer and regions to be stained were cut out and processed to visualise the ganglia.

The clearing and immunohistochemical method that was used has been optimised for gastrointestinal tissues that are investigated in thickmounts (Bossolani et al. 2018, Li et al. 2019).

*Quantitation of rat gastric enteric ganglia*

Z-stack tile scan images that were taken on an LSM800 confocal microscope were processed using Zen Blue software (Zeiss, Sydney, Australia) to ensure images were stitched together accurately before analysis was completed. The stitched file was opened in ImageJ (Fiji; <http://imagej.nih.gov/ij/)> and a background subtraction filter was applied and the file was converted to a tif image. Quantification was completed using Fiji v1.53c, with two additional plugins, 3D ImageJ Suite (Ollion et al., 2013) and MorphoLib (Legland et al. 2016)(available online).. For each image a specific threshold is set by the experimenter which accurately represents the immunoreactivity of the antibodies. A code was written to automate the image analysis. The script applies the given threshold and then applies filters to smooth and remove small particles from the image and generates a mask (or binary image) of the immunoreactivity selected., This mask contains 3 dimensional (3D)objects, representing any positive staining generated above the given threshold. Objects with greater than 20 µm distance separating them are defined as regions of interest (roi) representing individual ganglia. A unique intensity value is assigned to each ganglia roi, which can be used as an identifier. Ganglia roi were manually checked against the original image and assigned as either MP or SMP as defined by their position in the z stack. Two final masks are generated, a set of ganglia roi which most accurately represents all myenteric plexus (MP) ganglia and another for all the submucosa plexus (SMP) ganglia from the original image.

Ganglia roi could then be used in ImageJ to collect volume measurements (3D) from the z stack images, or to collect ganglia profile area measurements (2D) after projecting the z-stacks into a single image. The total area of the image was also measured for calculating ganglia density. Measurement data was then exported from ImageJ into excel and calculations were completed to determine ganglia areas and number of neurons per ganglia throughout all three regions of the stomach.

***Characteristics of Antibodies***

**Table 1.** Primary and secondary antibodies used in study and dilutions.

| Primary antibodies | | | | | |
| --- | --- | --- | --- | --- | --- |
| **Target** | **Antibody Code, reference** | **Source** | **Host**  **Species** | **Dilution** | **RRID** |
| nNOS | K205, (Herbison et al. 1996) (Williamson et al., 1996) | Gift; Dr P Emson | sheep | 1:1000 (sections); 1: 1000 (thick mounts ) | AB_2314960 |
| HuC/D | 16045 | Gift; Dr VA Lennon | Human | 1:4000-5000 (thick mounts) | AB_2314657 |
| α smooth muscle actin | AB569 | Abcam, Cambridge, UK | rabbit | 1:200 | AB_2223021 |
| Secondary antibodies | | | | | |
| **Target** | **Antibody Code** | **Source** | **Host**  **Species** | **Dilution** | **RRID** |
| Rabbit IgG | AB150070  Alexa Fluor® 555 | Abcam | Donkey | 1:1000 | AB_2783636 |
| Goat IgG | A32814  Alexa Fluor® 488 | Molecular Probes | Donkey | 1:500 | AB_2762838 |
| Human IgG | 709-585-149  Alexa Fluor® 594 | Jackson ImmunoResearch | Donkey | 1:250 | AB_2340572 |

The specificity of the K205 sheep anti-rat nNOS antibody was assessed using Western blotting and immune-absorption (Herbison et al. 1996). The antibody was found to recognize one main protein with a molecular mass of 155 kD in lanes containing the recombinant nNOS ( lane 1, Fig. 1) and rat hypothalamus. Other smaller protein bands were also present in both lanes and are likely to represent fragments of neuronal nNOS protein. To assess specificity in the immunocytochemical procedure, liquid phase adsorption experiments were undertaken by incubating the K205 at working dilution ( 1 : 5000) with recombinant nNOS ( 1 mM ) overnight at 4^o^C and then carrying out immunocytochemistry on preoptic area and mediobasal hypothalamic brain sections using the adsorbed antiserum in parallel with the normal K205. These experiments showed that all immunoreactivity was abolished by adsorption of the K205 antiserum with the recombinant neuronal nNOS protein.

**References**

Bossolani GDP, Pintelon I, Detrez JD, Buckinx R, Thys S, Zanoni JN, De Vos WH, Timmermans J-P (2018) Comparative analysis reveals Ce3D as optimal clearing method for in toto imaging of the mouse intestine. Neurogastroenterol Motil e13560:

Herbison AE, Simonian SX, Norris PJ, Emson PC (1996) Relationship of neuronal nitric oxide synthase immunoreactivity to GnRH neurons in the ovariectomized and intact female rat. J Neuroendocrinol 8:73-82

Li W, Germain RN, Gerner MY (2019) High-dimensional cell-level analysis of tissues with Ce3D multiplex volume imaging. Nature Protocols 14:1708-1733

Legland, D., Arganda-Carreras, I., & Andrey, P. (2016). MorphoLibJ: integrated library and plugins for mathematical morphology with ImageJ. Bioinformatics, 32, 2532-2534. doi:10.1093/bioinformatics/btw413

Ollion, J. Cochennec, F. Loll, C. Escudé, T. Boudier. (2013) TANGO: A generic tool for high-throughput 3D Image analysis for studying nuclear organization. Bioinformatics 2013: 29:1840-41.

Williamson S, Pompolo S, Furness JB (1996) GABA and nitric oxide synthase immunoreactivities are colocalized in a subset of inhibitory motor neurons of the guinea-pig small intestine. Cell Tissue Res 284:29-37
